# Multifactorial Comparative Proteomic Study of Cytochrome P450 2E1 Function in Chronic Alcohol Administration

**DOI:** 10.1371/journal.pone.0092504

**Published:** 2014-03-21

**Authors:** Yuan Wang, Yan Kou, Xiaodong Wang, Arthur Cederbaum, Rong Wang

**Affiliations:** 1 Department of Genetics and Genomic Sciences, Icahn School of Medicine at Mount Sinai, New York, New York, United States of America; 2 Department of Pharmacology and Systems Therapeutics, Icahn School of Medicine at Mount Sinai, New York, New York, United States of America; Indian Institute of Toxicology Research, India

## Abstract

With the use of iTRAQ technique, a multifactorial comparative proteomic study can be performed. In this study, to obtain an overview of ethanol, CYP2E1 and gender effects on liver injury and gain more insight into the underlying molecular mechanism, mouse liver proteomes were quantitatively analyzed using iTRAQ under eight conditions including mice of different genders, wild type versus CYP2E1 knockout, and normal versus alcohol diet. A series of statistical and bioinformatic analyses were explored to simplify and clarify multifactorial comparative proteomic data. First, with the Principle Component analysis, six proteins, CYP2E1, FAM25, CA3, BHMT, HIBADH and ECHS1, involved in oxidation reduction, energy and lipid metabolism and amino acid metabolism, were identified as the most differentially expressed gene products across all of the experimental conditions of our chronic alcoholism model. Second, hierarchical clustering analysis showed CYP2E1 knockout played a primary role in the overall differential protein expression compared with ethanol and gender factors. Furthermore, pair-wise multiple comparisons have revealed that the only significant expression difference lied in wild-type and CYP2E1 knockout mice both treated with ethanol. Third, K-mean clustering analysis indicated that the CYP2E1 knockout had the reverse effect on ethanol induced oxidative stress and lipid oxidation. More importantly, IPA analysis of proteomic data inferred that the gene expressions of two upstream regulators, NRF2 and PPARα, regulated by chronic alcohol feeding and CYP2E1 knockout, are involved in ethanol induced oxidative stress and lipid oxidation. The present study provides an effectively comprehensive data analysis strategy to compare multiple biological factors, contributing to biochemical effects of alcohol on the liver. The mass spectrometry proteomics data have been deposited to the ProteomeXchange with data set identifier of PXD000635.

## Introduction

Liver plays an essential role in ethanol metabolism.[1] Chronic consumption of alcohol can lead to fatty liver, alcoholic hepatitis and development of cirrhosis.[2]–[6] The pathogenesis of ethanol-induced liver injury is complex and involves, among other factors, gut-derived lipopolysaccharide, cytokines, the innate immune system, oxidative stress, as well as the interactions of these factors with intracellular signaling pathways.[7]–[9] A major molecular mechanism is the lipid peroxidation and oxidative stress induced by alcohol, which is a focus of considerable research.[10]–[13] Despite much research, the mechanisms by which alcohol causes cell injury are still not fully understand.

Alcohol is metabolized in hepatocytes through oxidation to acetaldehyde and subsequently from acetaldehyde to acetate as catalyzed by various enzymes or enzymatic systems, including the alcohol dehydrogenase and aldehyde dehydrogenase pathways, cytochrome P450 2E1 (CYP2E1) system and catalase.[14], [15] CYP2E1, which is up-regulated with chronic alcoholic ingestion, is an important source of reactive oxygen species (ROS) generation and contributor to oxidative stress in the liver.[16] Both cellular experiments and animal studies have demonstrated that CYP2E1 generates significant amount of ROS which participates in alcohol induced fatty liver.[15] However, understanding of key intracellular signaling pathways in which CYP2E1 contributes to the actions of ethanol still needs to be defined.

Gender differences of alcoholic liver injuries have been described previously,[17]–[19] but the underlying mechanisms are only partially characterized. For example, it is known that females develop alcoholic liver injury more rapidly and to a greater extent than males.[20] The increased susceptibility to alcohol-related liver injury in females has been postulated to be due to differences in first-pass metabolism in the stomach, different enzymatic activities in the liver, differences in ethanol distribution volumes in the body, differences in gut permeability to endotoxin and estrogen-induced increased susceptibility of Kupffer cells in the liver to gut-derived LPS.[21]

Several studies that investigated the rat liver proteomic profile differences with and without alcohol administration have been reported.[22]–[31] Using two-dimensional electrophoresis (2DE) based protein separation and quantification followed by matrix-assisted laser desorption ionization (MALDI)-mass spectrometry (MS) identification, about 150 significantly expressed proteins were reported in these studies. Few of these studies have been repeated or confirmed. In addition, 2DE-based proteomics has been used to identify differentially expressed proteins to predict the molecular basis for the observed gender susceptibility difference in an alcoholic steatohepatitis rat model.[31] The rapid development of stable isotope labeling methods used together with liquid chromatography and tandem mass spectrometry (LC-MS/MS), for protein identification and quantification, has significantly expanded the scale of proteomics studies. In combination with quantitative stable isotope labeling, mass spectrometry can be used to quantify and compare thousands of proteins from multiple samples. Isobaric Tag for Relative and Absolute Quantitation (iTRAQ) has gained popularity for its ability to perform concurrent identification and relative quantification of hundreds of proteins for up to 8 biological samples in a single experiment. With the use of this technique, a multifactorial comparative proteomic study can be investigated.

In the present study, a chronic alcoholism model was established using male and female, wild type and CYP2E1 knockout (KO) mice. To obtain an overview of both individual and combinatorial effects of ethanol, CYP2E1 and gender on liver injury, mouse liver proteomes were quantitatively analyzed using 8-plex iTRAQ reagents, under eight experimental conditions including mice of different genders, wild type versus CYP2E1 knockout, and normal versus alcohol diet. After protein identification, several statistical and bioinformatics approaches were applied to the protein expression data from different conditions, including principle component analysis, hierarchical clustering, analysis of variance (ANOVA), K-mean clustering and pathway mapping with Ingenuity Pathway Analysis (IPA), with the goal of elucidating the relationship among multiple factors upon alcohol consumption. The systematic analysis of protein expression in liver from mouse models confirmed the primary role of CYP2E1 in alcohol metabolism, and inferred potential upstream regulators. Therefore the present methodology provides a novel comprehensive interpretation of proteomics data and can be applied to other experimental settings involving multiple factors. We believe that the database generated by the present study could serve as a useful resource for studies of the mechanisms of alcohol induced liver injury.

## Materials and Methods

### Chemicals

Urea, 3-[(3-chloromidopropyl)dimethylammonio]-1-propanesulfonate (CHAPS), sodium fluoride (NaF), sodium orthovanadate (Na_3_VO_4_), ethylene diamine tetraacetic acid (EDTA), phenylmethanesulfonyl fluoride (PMSF) and alpha-cyano-4-hydroxycinnamic acid (CHCA) were purchased from Sigma-Aldrich (St. Louis, MO). Trifluoroacetic acid (TFA) and acetonitrile (ACN) were obtained from Thermo Scientific (Rockford, IL).

### Animals and ethanol administration

SV129 background CYP2E1 knockout mice were kindly provided by Dr. Frank J. Gonzalez (Laboratory of Metabolism, National Cancer Institute, Bethesda, MD) and breeding colonies established at Icahn School of Medicine Mount Sinai. SV129 wild type mice were purchased from Charles River Laboratory. The mice received humane care, and experiments were carried out according to the criteria outlined in the Guide for the Care and Use of Laboratory Animals and with approval of the Mount Sinai Institutional Animal Care and Use Committee. All mice were initially fed the control liquid dextrose diet (Bio-Serv, Frenchtown, NJ) for 3 days to acclimate them to Lieber and DeCarli liquid diets. Afterward, the mice were fed either the liquid ethanol diet or the control liquid dextrose diet for 4 weeks. The content of ethanol was gradually increased every 7 days from 10% (1.77%, vol/vol) of total calories to 20% (3.54%, vol/vol), 30% (5.31%, vol/vol), and finally 35% (6.2%, vol/vol) of total calories. The control mice were pair-fed with control dextrose diet on an isoenergetic basis. After 4 weeks, mouse serum and liver were collected. Liver was rapidly excised into fragments and washed with ice-cold saline. All samples were stored at −80°C. Liver sections were stained with H&E for pathological evaluation. CYP2E1 activity was measured by the rate of oxidation of p-nitrophenol to p-nitrocatechol by isolated hepatic microsomes.[32]

### Tissue sample preparation

Liver tissues were diced into pieces, and homogenized with a hand-held Dounce homogenizer in ice-cold homogenization buffer (40 mM Tris-HCl, 8 M urea, 4% CHAPS, 1 mM NaF, 1 mM Na_3_VO_4_, 2 mM EDTA, 1 mM PMSF, protease inhibitor cocktail (Roche, Indianapolis, IN), pH 7.4). The lysates were centrifuged at 20,000 *g* at 4°C for 30 min to remove any insoluble tissue debris. The supernatants were then collected and incubated with six volumes of cold acetone at −20°C overnight for protein precipitation. The precipitated proteins were centrifuged at 6,000 *g* at 4°C for 15 min. The pellets were collected and washed with ice-cold acetone twice, and then dried completely using a Savant SpeedVac (Thermo Scientific, Rockford, IL).

### Protein digestion and iTRAQ labeling

The protein pellets were solubilized in dissolution buffer (0.1% SDS in 500 mM triethylammonium bicarbonate, pH 8.0). The samples were sonicated with a probe sonicator (Thermo Scientific, Rockford, IL) on ice for 10 sec, 3 times followed by centrifugation at 35,000 *g* at 4°C for 30 min. The supernatants were collected and the total protein concentrations were measured by the Bradford assay (Thermo Scientific, Rockford, IL). 200 μg of protein from each sample was reduced with 5 mM tris-(2-carboxyethyl) phosphine at 60°C for 1 hr, alkylated with 10 mM S-methyl methanethiosulfonate (MMTS) at room temperature for 10 min and digested with trypsin 1∶20 (E/S, w/w) at 37°C for 18 hrs. Subsequently, each tryptic digest was labeled for one hour with one of the eight iTRAQ reagents according to the manufacturer's protocol (AB Sciex, Foster City, CA): Tag_113_, WT/dextrose diet/female; Tag_114_, WT/dextrose diet/male; Tag_115_, WT/ethanol diet/female; Tag_116_, WT/ethanol diet/male; Tag_117_, CYP2E1 KO/dextrose diet/female; Tag_118_, CYP2E1 KO/dextrose diet/male; Tag_119_, CYP2E1 KO/ethanol diet/female; Tag_121_, CYP2E1 KO/ethanol diet/male mice. These eight iTRAQ-derivatized samples were pooled and then desalted using a Sep-Pak cartridge (Waters, Milford, MA). Peptides were eluted with 0.1% acetic acid in 50% ACN. The peptide mixture was then dried completely using a Savant SpeedVac.

### Peptide OFFGEL fractionation

3100 OFFGEL Fractionator and OFFGEL kit pH 3–10 (Agilent Technologies, CA, USA) with 24 wells setup were used. Peptides were diluted in 3.6 mL of the OFFGEL solution buffer without glycerol in deviation from the supplier's protocol. IPG strips were rehydrated by adding 40 μL of OFFGEL solution buffer per well for 15 min. Then, 150 μL of sample was loaded in each well. Peptide focusing was performed until the voltage-hour reached 50 kVh with a maximum voltage of 8,000 V and maximum current of 50 μA. After focusing, the 24 peptide fractions were transferred into new set of Eppendorf tubes and wells were rinsed with 200 μL of water for 15 min and pooled together with peptide fractions. The peptide fractions were then concentrated in a SpeedVac prior to LC-MS/MS analysis.

### Nano-LC separation

The dried peptides were re-dissolved in 0.1% TFA, 2% ACN in water (v/v). One third of each peptide fraction was further separated using an Ultimate nano LC system (Dionex, Sunnyvale, CA) equipped with a C18 trap (5.0 mm×300 μm ID, LC Packings) and a 15 cm×100 μm ID column packed in-house with 5 μm Magic C18 beads (Michrom Bioresources, Auburn, CA) at a flow rate of 700 nL/min. Solvent A was 2% ACN in water with 0.1% TFA, and solvent B was 98% ACN in water with 0.1% TFA. The peptides were desalted for 5 min using only solvent A on the trap column, followed by a separation using the following gradient: 0 to 10% B in 10 min, 10% to 50% B in 55 min, and 50% to 90% B in 5 min. Chromatograms were recorded at the wavelength of 214 nm. Peptide fractions were collected using a modified Probot microfraction collector (Dionex) with a voltage pulser. Column effluent was mixed with MALDI matrix, 5 mg/ml CHCA in 90% ACN, 0.1% TFA, and collected at a frequency of one spot every 20 s on an Opti-TOF LC/MALDI insert blank plate (AB Sciex).

### Mass spectrometry analysis and database search

MALDI plates were analyzed with a TOF/TOF 5800 mass spectrometer (AB Sciex). The instrument was calibrated using the 4700 mass calibration standard. MS spectra between *m/z* 800 and 4,000 were acquired for every spot using 1,000 laser shots in reflector mode. The 20 most intense ion signals per spot having a S/N>10 were selected as precursors for MS/MS acquisition using 2,000 laser shots. Peptide and protein identifications were performed with the ProteinPilot Software 4.5 (AB Sciex) using the Paragon algorithm. Combined data and spectra from all 24 OFFGEL fractions were searched against the UniProt mouse database (release-2010_11). The following search parameters were selected: iTRAQ 8-plex peptide label, cysteine alkylation, trypsin specificity, ID focus on biological modifications, and processing including quantitation and thorough ID. A protein with a confidence threshold of 95% (unused confidence threshold ProtScore>1.3) was reported and the corresponding False Discovery Rate (FDR) was less than 1%. In protein grouping, competitor threshold was set as 2.00 in ProtScore.

### Clustering and statistical analysis

Functional annotation of protein was conducted using DAVID Bioinformatics Resources 6.7 (NIAID/NIH) in reference of Toppgene (http://toppgene.cchmc.org/), Protein Knowledgebase (UniProtKB) and relevant literature in PubMed. In this study, protein ratio and p-value reported by ProteinPilot were used for quantitative analysis. Protein ratio (R) was transformed to log2 R. Proteins with |log2 R|>1 and p<0.05 were defined as significantly changed proteins.

The principle component analysis (PCA) was applied to the log2 R of proteins with p<0.05 in at least one of the seven experimental observations, using princomp function in Matlab (MathWorks, Natick, MA, version R2011b). With the PCA results, proteins were further ranked according to the Hotelling's T^2^ test.

To compare the overall protein expression level in seven observations, analysis of variance (ANOVA) was conducted on the same data set as PCA with the null hypothesis that the average protein expression change is equal in seven selected observations. In order to find the significantly different observation(s), multiple comparison procedure was implemented in Matlab with multicompare function using Tukey-Kramer correction method.

Hierarchical clustering was applied to proteins with p-value less than 0.05 in at least one observation. An expression value which is considered as no significant change in protein expression (p>0.05) was assigned to missing data point. The Euclidean distance was applied to the log2 R, and linkage type is “complete”. All expression value was normalized within observation before calculating the distance. The Matlab function clustergram was used for hierarchical clustering.

As for K-mean clustering, two observations were selected based on ANOVA multiple comparison results. In this analysis, only significantly changed proteins in both observations were analyzed. We used the deviation from equal expression as distance for K-mean clustering, which is defined as below:
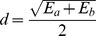
where *E_a_*, *E_b_* is the log2 R of protein expression change in observation *a* and *b*, respectively. Three clusters were set to be searched for using K-mean clustering algorithm (function kmeans) in Matlab.

### Ingenuity pathways analysis

The quantitative data were analyzed using Ingenuity Pathways Analysis (IPA) (Ingenuity Systems, www.ingenuity.com) for molecular pathway and network analysis of significantly changed proteins. Each protein identifier with the quantitative information was uploaded and mapped to its corresponding object in Ingenuity's Knowledge Base to algorithmically generate molecular networks. In the networks reported below, molecules are represented as nodes, and their relationship is denoted as an edge.

### Western blot analysis

Equal aliquots of total proteins were loaded and separated on 4–12% NuPAGE Bis-Tris gels (Invitrogen, Cartsbad, CA) and then electrotransferred onto nitrocellulose membranes. The polyclonal anti-CA3, anti-BHMT, anti-HIBADH, anti-ECHS1, and anti-ACOX1 antibodies were purchased from Proteintech (Chicago, IL); anti-PPARα and anti-NRF2 polyclonal antibodies were obtained from Santa Cruz (Santa Cruz, CA). The monospecific anti-CYP2E1 antibody was a gift from Dr. Jerome Lasker, Hackensack Biomedical Research Institute, Hackensack, NJ. The monoclonal anti-β actin antibody was purchased from Sigma-Aldrich (St. Louis, MO). Secondary antibodies conjugated with horseradish peroxidase were used to amplify the immuno-recognition signals with the peroxidase activity monitored using an ECL kit (Thermo Scientific, Pittsburgh, PA). The images were captured with FluorChem Q (ProteinSimple, San Jose, CA).

## Results and Discussion

### Comparison of the effect of chronic ethanol feeding on liver to body weight ratio and steatosis in wild-type and CYP2E1 knockout mice

**Figure 1:**
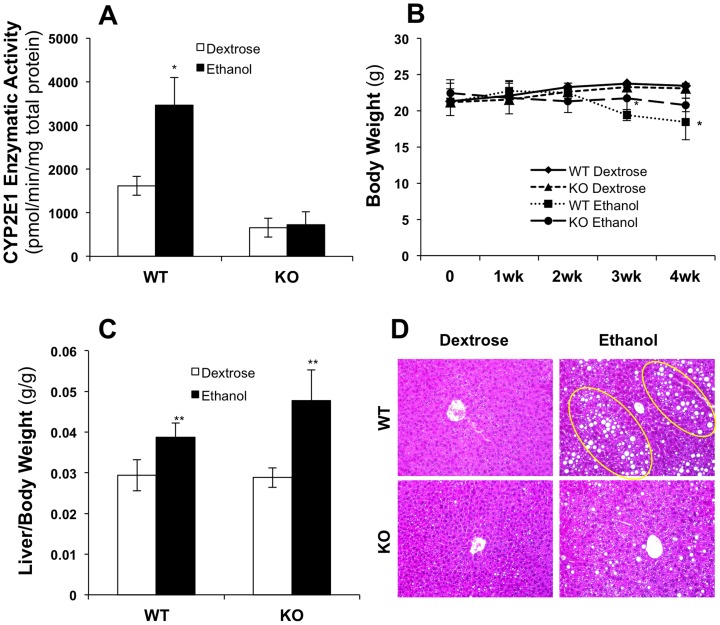
Mouse model after chronic ethanol feeding. (A) Microsomal p-nitrophenol hydroxylase activities; (B) Mouse body weight measurement; (C) Liver to body weight ratio; (D) Mouse liver tissue specimens stained with hematoxylin and eosin (H&E), arrows showing lipid droplets. *p<0.05 and **p<0.01, compared with WT dextrose group. (n = 4 pairs of mice in each group)

As expected, CYP2E1 protein was absent in CYP2E1 knockout mice, as reflected by the decreased enzymatic activities of CYP2E1 measured by the rate of oxidation of p-nitrophenol to p-nitrocatechol by isolated hepatic microsomes (Fig. 1A). It is known that CYP2E1 can be induced by ethanol. In our chronic alcohol-feeding model, CYP2E1 enzymatic activity was significantly increased (about 2-fold) in wild-type mice, but activity was low and not changed in CYP2E1 knockout mice (Fig. 1A). After 4 weeks of ethanol feeding, serum ethanol levels increased from 1.78±0.33 μmol/ml to 6.60±0.97 μmol/ml in wild-type mice and from 2.39±0.29 μmol/ml to 6.37±0.89 μmol/ml in CYP2E1 knockout mice, which did not differ significantly between the knockout and wild-type mice. Body weight did not change during the first 2 weeks of ethanol feeding in wild-type or knockout mice (Fig. 1B). In the third week, ethanol-fed wild-type mice had lost some weight compared with the dextrose-fed mice. After 4 weeks of ethanol feeding, the body weight of the wild-type mice was further decreased. The ethanol-fed CYP2E1 knockout mice lost a slight but not significant amount of body weight, compared with the dextrose-fed knockout mice (Fig. 1B). The liver to body weight ratio was elevated by ethanol in knockout and wild-type mice significantly (Fig. 1C). Furthermore, after 4 weeks of ethanol feeding, extensive lipid droplets were observed in the wild-type mice, but only a small number of tiny lipid droplets were observed in the knockout mice (Fig. 1D).

### The major identified proteins are mitochondrial proteins and catalytic enzymes

A total of 878 proteins were identified with local false discovery rate (FDR) less than 1% and among which about 98.3% (863/878) were quantified. Detailed information on identified proteins is provided in Table S1. The cellular components and molecular functions of all identified proteins were annotated based on Gene Ontology (GO) using DAVID. The list of 878 gene symbols input returned 836 DAVID Gene ID. Protein cellular component analysis (GOTERM_CC_FAT) showed 34.1% of the identified proteins, 285 of them, are localized in mitochondria. Among others, 9.6% (80 proteins) localized in cytosol, 2.9% (24 proteins) localized in endoplasmic reticulum (ER), and 3.0% (25 proteins) localized in peroxisomes. Biological process annotation of this data (GOTERM_BP_FAT) indicated 163 proteins (19.5%) are involved in oxidation reduction, 52 proteins (6.2%) are cofactors of metabolic processes, 22 (2.6%) and 32 (3.8%) of them are involved in alcohol catabolic process and fatty acid metabolic processes, respectively. GO molecular function (GOTERM_MF_ALL) classification indicated that about half of the identified proteins (426 of them) have catalytic activities (50.9%). Many of the identified proteins were annotated having oxidoreductase activities, 38 of them (4.5%) acting on CH-OH group of donors; 33 of them (3.9%) acting on the CH-OH group of donors, NAD or NADP as acceptor; 20 of them (2.4%) acting on NADH or NADPH; and 18 of them (2.2%) acting on the aldehyde or oxo group of donors, respectively. In addition, 14 identified proteins (1.7%) have antioxidant activities and 13 proteins (1.6%) have glutathione transferase activities. All of the above analyses indicated that the quantitative proteome data generated here could allow investigation of the effects of chronic alcohol consumption on mitochondrial functions and catalytic enzyme expressions, especially those involved in oxidation reduction and alcohol metabolism. With our experimental design, using wild type versus CYP2E1 knockout mice in different genders and fed with normal versus alcohol diet, we were able to reveal the effects of CYP2E1 and the gender difference on ethanol induced changes at a proteomic level.

### The most differentially expressed proteins in this multivariate proteomic analysis

**Figure 2:**
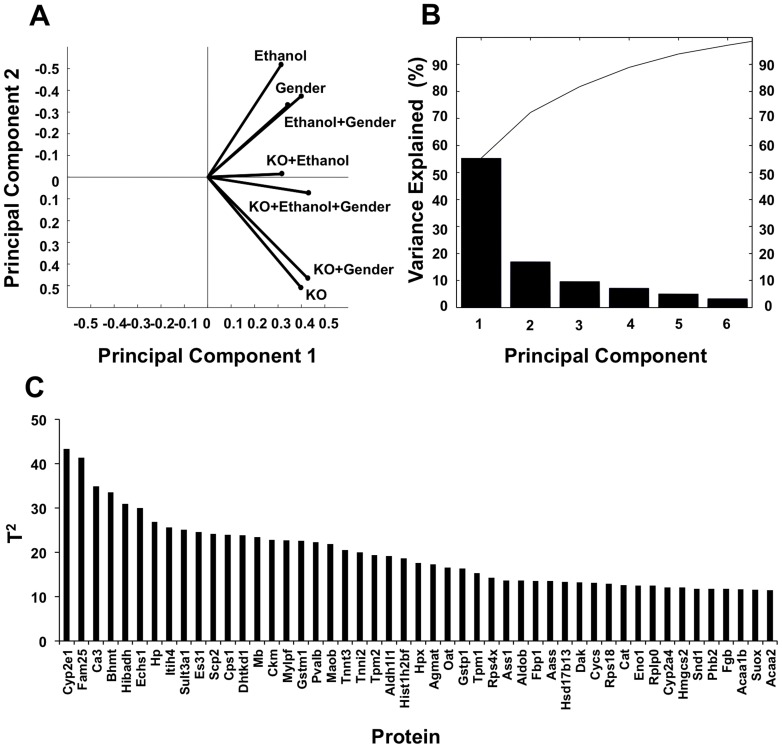
Principal component analysis of the proteomic results. (A) Biplots of PCA of seven observations which predicted to the space defined by the first and second principle components. (B) Variance explained by the top six principle components in seven observations. Each bar represents the individual variance explained by the principle component, and the curve shows cumulative explained variance of top principle components. (C) Proteins having Hotelling's T^2^ values greater than the third quartile.

**Figure 3:**
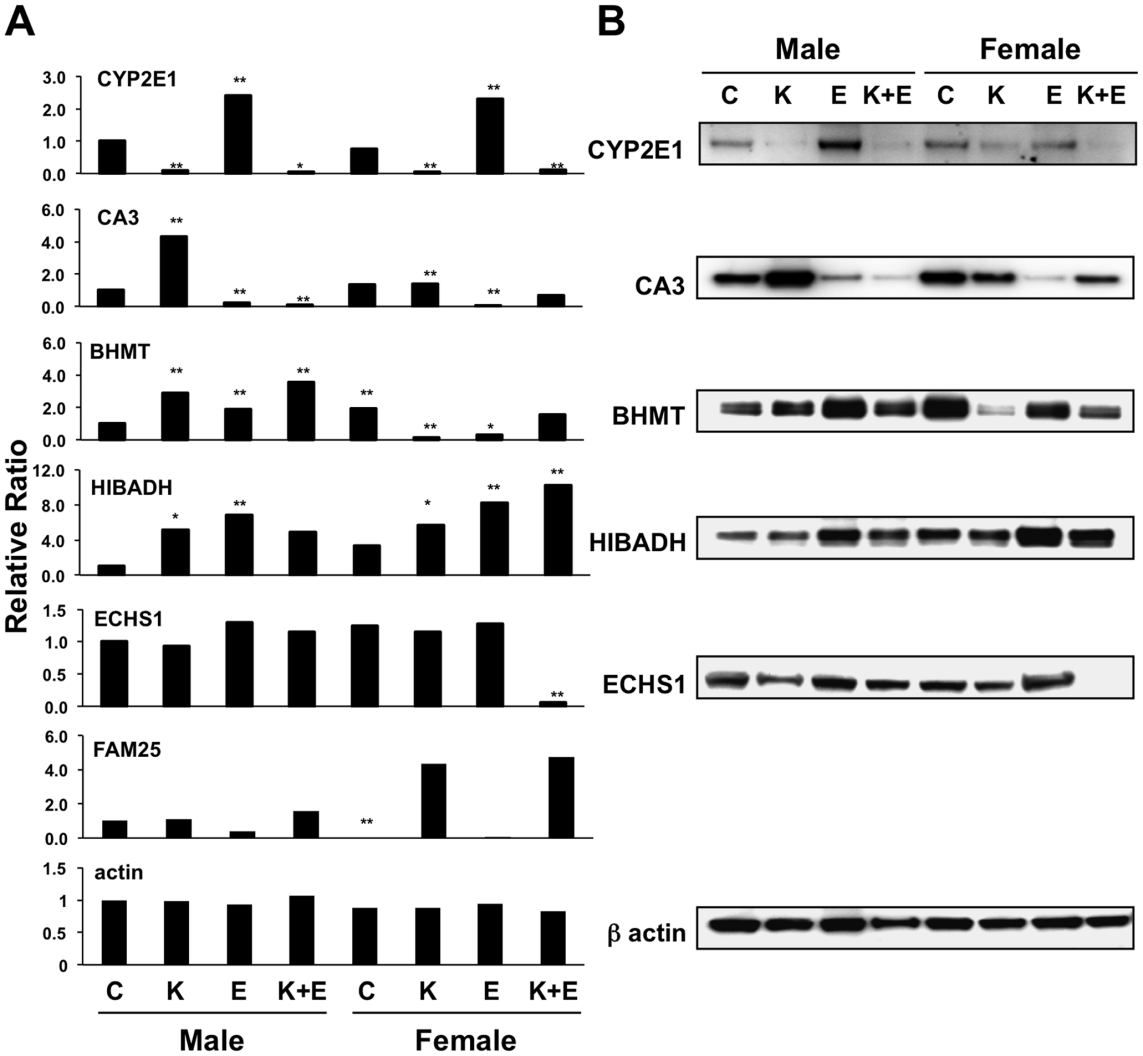
Quantitative analysis of the top six proteins according to Hotelling's T^2^ test, CYP2E1, CA3, BHMT, HIBADH, ECHS1 and FAM25 in the eight mice models. (A) iTRAQ labeling mass spectrometry results of the top six proteins. All values are relative to control mice (Dextrose diet, wild-type, male mice), *p<0.05 and **p<0.01. (B) Western blot of the same six proteins except FAM25 (no commercial antibody available). β-Actin was used as the loading control. C, control; K, CYP2E1 knockout; E, ethanol.

To evaluate the multifactorial conditions and reduce the complexity of the proteomic data, principal component analysis (PCA) analysis was conducted with Matlab statistical software. Wild-type male mice fed with dextrose diet was selected as control to be compared to seven observations, protein expression ratio of CYP2E1 knockout male mice fed with dextrose diet (R_KO_, 118/114), wild-type male mice fed with ethanol (R_E_, 116/114), CYP2E1 knockout male mice fed with ethanol (R_KO+E_, 121/114), wild-type female mice fed with dextrose diet (R_F_, 113/114), CYP2E1 knockout female mice fed with dextrose diet (R_F-KO_, 117/114), wild-type female mice fed with ethanol (R_F-E_, 115/114), and CYP2E1 knockout female mice fed with ethanol (R_F-KO+E_, 119/114) were analyzed. All identified proteins with quantitative data were tested with p<0.05 in at least one of the seven experimental conditions (observations). This test resulted in 270 proteins that were defined as expression changed proteins. PCA was conducted on the 270 expression changed proteins. The biplot of the first two principal components analysis is shown in Fig. 2A. In this biplot, the seven observations are projected to the space defined by the first and second principle components. The first two principal components can account for approximately 70% variance in the entire dataset and clearly grouped the seven observations into three clusters. This grouping indicated that the variances brought by the CYP2E1 knockout and chronic ethanol feeding were more significant over those caused by gender. The Scree plot of the PCA showed that the percentage of variances explained by the first six principal components could explain more than 90% of the variance in the entire dataset (Fig. 2B). To further identify the most representative proteins of the total variation in the seven observations, Hotelling T^2^ test was conducted post the PCA. The list of the protein having T^2^ values greater than the third quartile T^2^ are shown in Fig. 2C. The T^2^ value is a statistical measure indicating the multivariate distance of each protein from the center of the dataset.[33] The top six proteins according to Hotelling's T^2^ test, CYP2E1, FAM25, Carbonic anhydrase 3 (CA3), Betaine-homocysteine S-methyltransferase 1 (BHMT), 3-hydroxyisobutyrate dehydrogenase (HIBADH) and Enoyl-CoA hydratase, mitochondrial (ECHS1), are therefore pointed as the most extremely differentially expressed proteins across the seven observations. The quantitative mass spectrometry data as well as Western blot results of these proteins, except for FAM25, which has no gene annotation and without a commercial antibody currently available, showed that the expressions of these six proteins are dramatically different in the eight animal models (Fig. 3A and 3B). For examples, the expression of CYP2E1 was diminished in the knockout mice, but its expression was greatly induced by ethanol in the wild-type mice as previously reported.[16], [34] The expressions of CA3 were greatly reduced with ethanol feeding but significantly increased when CYP2E1 was knocked out in both genders. The level of ECHS1 was significantly reduced in female CYP2E1 knockout mice fed with ethanol. However, FAM25, which is composed of 89 amino acids, was only reduced in wild-type female mice (log2 R_F_ -6.09, p = 0.017). These proteins mediate biological functions such as oxidation reduction (CYP2E1 and HIBADH), one-carbon metabolism (BHMT and CA3) and lipid metabolism (ECHS1). Three of these proteins, CYP2E1 (found also in the endoplasmic reticulum), ECHS1, and HIBADH are localized in mitochondria. Protein-protein interaction network analysis by Ingenuity revealed these proteins are involved in different networks. These results indicate that although oxidative stress is a major component for alcohol damage in liver, a much wider array of cellular functions could also be affected by chronic ethanol administration.

### Chronic alcohol effects on liver proteomes in different genders

To analyze the effects of chronic ethanol feeding on the liver proteome, protein expression changes of ethanol-fed versus dextrose-fed wild type male mice (log2 R_E_, 116/114) were analyzed. According to the aforementioned criteria (|log2 R|>1 and p<0.05), 35 proteins were identified as significantly changed after chronic ethanol feeding. 29 of them were increased and 6 of them were decreased (Table 1 and Table S2). Among the up-regulated proteins, 10 are involved in oxidative stress (GSTM1, HIBADH, ALDH1L1, CYP2A4, AASS, ASS1, POR, CYP2E1, SUOX and MDH2), 4 are involved in alcohol metabolism (ALDH2, ALDH1B1, DHTKD1 and ALDOB), 9 are involved in fatty acid and amino acid metabolism (CPS1, FTCD, ACAA2, ACSM, ACSL1, SHMT2, GUL1, OAT and GPT), and 2 are involved in the developmental process (KRT8 and TNNI2). In addition, two heat shock proteins (HSPA9 and HSPD1), one serum carrier protein (TF), and one energy production protein (PCX), were also increased after chronic alcohol feeding. On the other hand, only 6 proteins were down-regulated, including ENO1 (alcohol metabolism), CA3 and SCP2 (fatty acid and amino acid metabolisms), RPS2 and HIST1H2BF (DNA binding) and VIM (developmental process). Furthermore, of the above 35 proteins, expressions of 16 of them have been reported to be altered by alcohol administration in previous studies (marked with ** in Table 1),[14], [35]–[38] of which, 10 proteins were reported altered by other proteomic experiments (marked with * in Table 1).[22], [23], [25], [27]–[31], [39] The other 19 proteins reported for the first time to be affected by alcohol administration are a result from this study (Table 1).

**Table 1:** Proteins significantly changed after ethanol administration in male and female mice.

Accession Number	Protein Name	Gene Symbol	Function	log_2_(116∶114)	log_2_(115∶113)
B2KGV2	Carbamoyl-phosphate synthase	Cps1	fatty acid metabolism	3.11	-
A2ATU0	Probable 2-oxoglutarate dehydrogenase E1	Dhtkd1	alcohol metabolism	3.1	-
P10649	Glutathione S-transferase Mu 1	Gstm1*	oxidation reduction	2.92	1.37
A0ZNJ2	3-hydroxyisobutyrate dehydrogenase	Hibadh*	oxidation reduction	2.76	-
Q8R0Y6	10-formyltetrahydrofolate dehydrogenase	Aldh1l1**	oxidation reduction	2.55	-
Q3UER1	Fructose-bisphosphate aldolase	Aldob*	alcohol metabolism	2.09	-
Q91XG2	Cytochrome P450, family 2A4	Cyp2a4**	oxidation reduction	2.07	-
Q3UWN2	Putative uncharacterized protein	Aass	oxidation reduction	2.01	-
Q7TSZ0	Heat shock protein 9	Hspa9*	chaperone	2.01	-
Q3UJ34	Argininosuccinate synthase	Ass1**	oxidation reduction	1.95	-
Q3UKT3	Putative uncharacterized protein	Oat	amino acid metabolism	1.87	-
Q544B1	Aldehyde dehydrogenase 2	Aldh2*	alcohol metabolism	1.79	-
Q546G4	Albumin 1	Krt8*	developmental process	1.63	2.07
Q3TCQ3	Putative uncharacterized protein	Pcx	energy production	1.57	-
A2A6J8	Troponin I, skeletal, fast 2	Tnni2	developmental process	1.57	2.21
Q91XD4	Formimidoyltransferase-cyclodeaminase	Ftcd	fatty acid metabolism	1.45	−1.69
P37040	NADPH—cytochrome P450 reductase	Por**	oxidation reduction	1.44	-
B1AWX7	Aldehyde dehydrogenase 1B1	Aldh1b1	alcohol metabolism	1.42	-
Q566C3	Alanine aminotransferase 1	Gpt	amino acid metabolism	1.4	-
Q3TIT9	Acetyl-Coenzyme A acyltransferase 2	Acaa2	fatty acid metabolism	1.37	-
Q921I1	Serotransferrin	Tf*	serum carrier protein	1.37	1.9
D3Z106	Uncharacterized protein	Acsm1	fatty acid metabolism	1.36	-
Q6GTG6	Long-chain-fatty-acid—CoA ligase 1	Acsl1	fatty acid metabolism	1.29	-
Q05421	Cytochrome P450 2E1	Cyp2e1**	oxidation reduction	1.26	1.59
Q8R086	Sulfite oxidase, mitochondrial	Suox	oxidation reduction	1.24	-
P63038	60 kDa heat shock protein	Hspd1*	chaperone	1.14	-
P08249	Malate dehydrogenase	Mdh2**	oxidation reduction	1.06	1.49
Q9CZN7	Serine hydroxymethyltransferase	Shmt2	fatty acid metabolism	1.02	-
P15105	Glutamine synthetase	Glul	fatty acid metabolism	1.01	-
Q3TXS9	Putative uncharacterized protein	Rps2	DNA binding	−1.29	−2.51
Q3U6S1	Putative uncharacterized protein	Vim	developmental process	−1.55	−1.38
Q5FW97	Enolase	Eno1*	alcohol metabolism	−1.83	−1.17
P32020	Non-specific lipid-transfer protein	Scp2	fatty acid metabolism	−1.97	-
P10853	Histone H2B type 1-F	Hist1h2bf*	DNA binding	−1.98	−3.89
P16015	Carbonic anhydrase 3	Ca3*	fatty acid metabolism	−2.14	−4.98
Q91W60	Inter alpha-trypsin inhibitor, heavy chain 4	Itih4	metabolism	-	4.33
Q545Y3	Putative uncharacterized protein	Tpm1	developmental process	-	2.99
A2A4Z2	Troponin C2, fast	Tnnc2	developmental process	-	2.92
Q8CF02	Protein FAM25	Fam25	N/A	-	2.29
Q02819	Nucleobindin-1	Nucb1	DNA binding	-	2.01
P56135	ATP synthase-coupling factor 6	Atp5j	energy production	-	1.93
P63242	Eukaryotic translation initiation factor 5A-1	Eif5a	apoptosis process	-	1.85
Q5FWJ5	Hnrpk protein	actg1-b	DNA binding	-	1.83
Q8C7E7	Starch-binding domain-containing protein 1	Stbd1	amino acid metabolism	-	1.71
Q8VDD5	Myosin-9	Myh9*	developmental process	-	1.65
Q58E70	Putative uncharacterized protein	Krt8*	developmental process	-	1.54
P27773	Protein disulfide-isomerase A3	Pdia3*	oxidation reduction	-	1.51
Q3UKP2	Hemopexin, isoform CRA_f	Hpx	signaling/transcription	-	1.47
Q91XF8	Apolipoprotein A-IV	Apoa4*	fatty acid metabolism	-	1.46
Q544Y7	Cofilin 1, non-muscle	Cfl1	developmental process	-	1.41
Q4KL76	Heat shock protein 1 (Chaperonin 10)	Hspe1	chaperone	-	1.4
Q922C8	Prolyl 4-hydroxylase, beta polypeptide	P4hb*	oxidation reduction	-	1.36
Q3UEK9	Alpha-2-HS-glycoprotein, isoform CRA_a	Ahsg	developmental process	-	1.32
Q99K47	Fibrinogen, alpha polypeptide	Fga	signaling/transcription	-	1.28
P19157	Glutathione S-transferase P 1	Gstp1	oxidation reduction	-	1.28
E0CXN5	Uncharacterized protein	Gpd1	oxidation reduction	-	1.25
Q9CQB4	MCG67985	Uqcrb*	oxidation reduction	-	1.22
A8DUK0	Beta-globin	Hbb-b1*	transport	-	1.2
Q4FJX9	Superoxide dismutase	Sod2*	oxidation reduction	-	1.1
Q54AH9	Beta-2-globin (Fragment)	Hbb*	transport	-	1.08
B2RXY7	Carbonyl reductase 1	Cbr1	oxidation reduction	-	1.02
Q9DCY1	Peptidyl-prolyl cis-trans isomerase	Ppib*	protein folding	-	−1.02
Q91V38	Heat shock protein 90, beta (Grp94)	Hsp90b1*	chaperone	-	−1.06
Q3TZJ3	Putative uncharacterized protein	Hspa8*	chaperone	-	−1.16
D2KHZ9	Glyceraldehyde-3-phosphate dehydrogenase	Gapdh*	oxidation reduction	-	−1.28
P62264	40S ribosomal protein S14	Rps14	signaling/transcription	-	−1.3
Q3TDN8	Putative uncharacterized protein	Bphl	signaling/transcription	-	−1.34
P14152	Malate dehydrogenase	Mdh1	oxidation reduction	-	−1.37
Q9JMH6	Thioredoxin reductase 1	Txnrd1	oxidation reduction	-	−1.37
P49429	4-hydroxyphenylpyruvate dioxygenase	Hpd	oxidation reduction	-	−1.58
O88844	Isocitrate dehydrogenase [NADP]	Idh1*	oxidation reduction	-	−1.63
Q3V2F7	Fatty acid binding protein 1, liver	Fabp1*	fatty acid metabolism	-	−1.74
Q3U9G2	Putative uncharacterized protein	Hspa5	chaperone	-	−1.75
Q3TY87	Putative uncharacterized protein	Fah*	amino acid metabolism	-	−1.77
Q99JY0	Trifunctional enzyme subunit beta	Hadhb	oxidation reduction	-	−1.78
Q4FZE6	Putative uncharacterized protein	Rps7	signaling/transcription	-	−1.9
P05784	Keratin, type I cytoskeletal 18	Krt18*	developmental process	-	−1.97
P17563	Selenium-binding protein 1	Selenbp1*	signaling/transcription	-	−1.98
Q5EBH4	Dimethylglycine dehydrogenase	Dmgdh	oxidation reduction	-	−2.06
Q99KR3	Beta-lactamase-like protein 2	Lactb2	fatty acid metabolism	-	−2.21
Q53ZU7	Peroxiredoxin 6	Prdx6*	oxidation reduction	-	−2.3
Q5M9M5	MCG10806	Rpl23a	DNA binding	-	−2.62
O35459	Betaine—homocysteine S-methyltransferase 1	Bhmt*	amino acid metabolism	-	−2.74
Q3UIA9	Fumarate hydratase 1	Fh1	fatty acid metabolism	-	−2.87
Q63880	Liver carboxylesterase 31	Es31	alcohol metabolism	-	−3.19
Q56A15	Cytochrome c, somatic	Cycs	energy production	-	−3.22
Q3V235	Prohibitin 2	Phb2*	DNA binding	-	−3.22
P67778	Prohibitin	Phb*	DNA binding	-	−3.43
P56480	ATP synthase subunit beta	Atp5b*	energy production	-	−3.6
Q03265	ATP synthase subunit alpha	Atp5a1*	energy production	-	−3.79

NOTE. This table contains the 90 liver proteins that display more than 2.0 fold change either in ethanol treated male mice or female mice. For more detailed information of these proteins, please refer to Table S2.

*The proteins reported by previous proteomic studies.

** The proteins reported by previous studies.

Similarly, analyzing protein expression changes of ethanol-fed versus dextrose-fed wild type female mice (log2 R_E-F_, 115/113), 67 proteins were identified as being significantly changed (Table 1 and Table S2). Among them, 32 were up-regulated and 35 were down-regulated. Among these proteins, 29 proteins have been previously shown to be altered after ethanol feeding in male mice in other proteomic studies (marked with *).[22]–[31], [40]

Comparing the differentially expressed proteins between male and female mice in response to chronic ethanol feeding, 12 proteins were changed in both genders while 23 and 55 proteins were only changed in male mice or female mice, respectively. Among the 23 unique proteins to male mice, 22 of them were up-regulated and 1 was down-regulated. Regarding the 55 proteins uniquely changed in female mice, 26 of them were up-regulated and 29 of them were down-regulated. Of the shared 12 proteins, only one protein, formimidoyltransferase-cyclodeaminase (FTCD), showed expression changes in opposite directions, being elevated in male mice and decreased in female mice. FTCD is a folate-dependent bifunctional enzyme involved in the histidine-degradation pathway. It catalyzes one-carbon units transfer from formiminoglutamate, a metabolite in the histidine degradation pathway, to tetrahydrofolate, reversibly as well as catalyzes deamination of 5-formimidoyltetrahydrofolate. FTCD is also a liver-specific autoantigen in patients with autoimmune hepatitis.[41], [42] It was identified in a proteomic study as being down-regulated in hepatocellular carcinoma (HCC).[43] It has been reported that serum folate levels were within the normal range but lower in alcohol liver disease (ALD) patients and actively drinking subjects compared to healthy subjects.[44] The review by Medici summarized the mechanisms for this observation that include DNA damage with strand breaks, oxidation, and apoptosis occurring in experimental ALD in association with decreased S-adenosylmethionine (SAM) levels.[45] Therefore the discrepant FTCD level between male and female mice observed in our study could to be an explanation for the higher increased progression of ALD which occurs in females after alcohol consumption.

**Figure 4:**
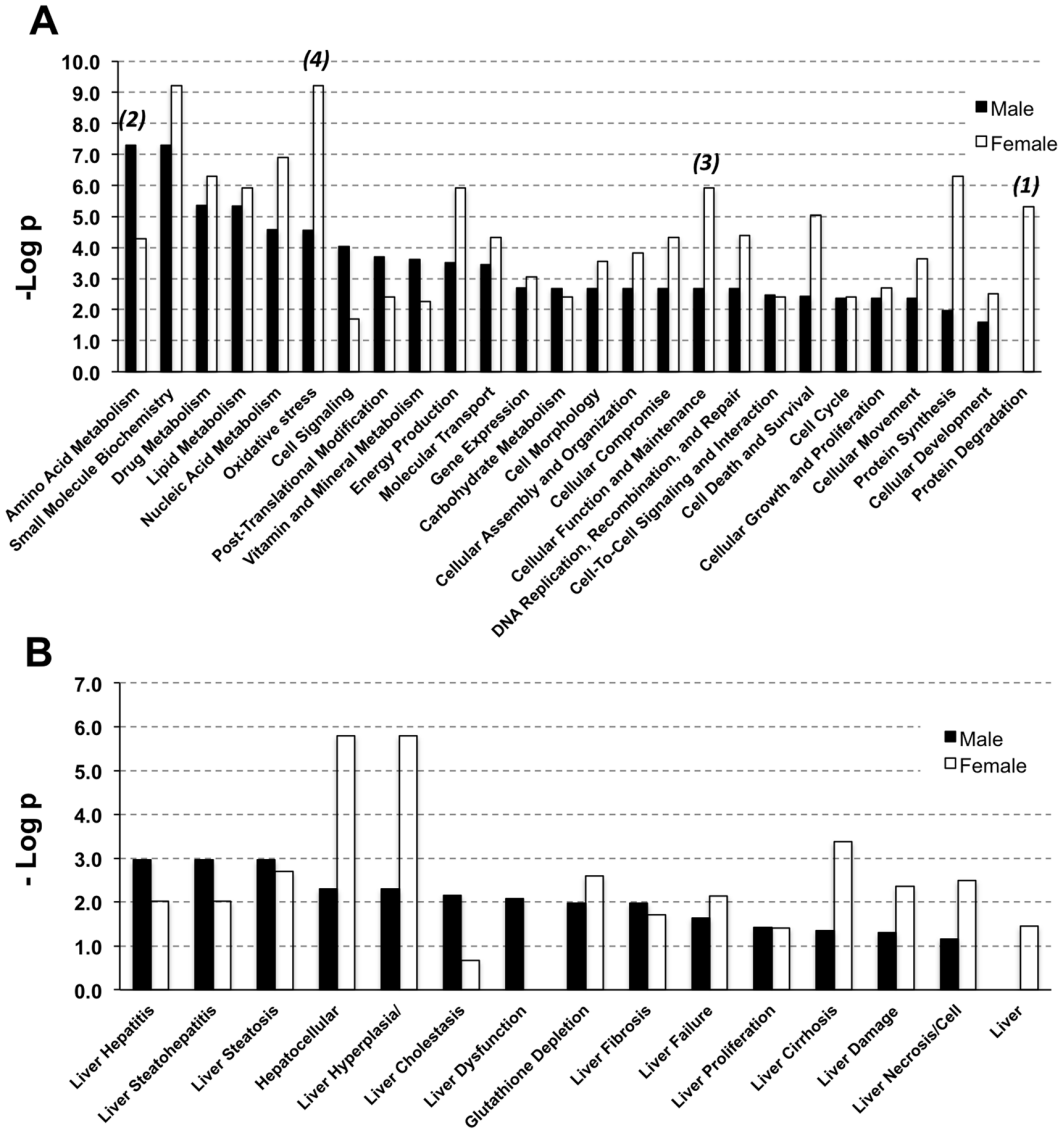
Gender differences in response to chronic alcohol feeding summarized by IPA analysis with the significantly changed proteins in male and female mice. (A) Molecular and cellular function differences after chronic ethanol feeding in male and female mice. (B) Hepatotoxicity differences after chronic ethanol feeding in male and female mice.

Compared to male mice, four aspects exhibited differences in the ethanol-fed female mice (Fig. 4A): 1) Heat shock proteins (HSP90B1, HSPD1, HSPA5, HSPA8 and HSPA9) were decreased in female mice, but not males. HSPs as molecular chaperone proteins, participate in the folding of newly synthesized proteins, unfolding, aggregation, as well as transportation and degradation of proteins.[46] HSPD1 is expressed in response to oxidative stress.[47] Increased expression of HSPD1 has been reported in various inflammatory diseases, such as rheumatoid arthritis, insulitis, and atherosclerosis.[48]–[50] Although the precise mechanism for lower expression of HSPs in females after chronic ethanol administration has not been clarified, the down-regulation of HSPs as shown in this study may point to a lower adaptive and protective level in the liver which may contribute to the higher susceptibility to alcohol related liver injury in females (Fig. 4B). 2) Proteins involved in fatty acid and amino acid metabolism, alcohol metabolism and energy production, such as FABP1, LACTB2 BHMT and ATP synthase, were up-regulated in male mice fed with alcohol compared with control but these proteins neither showed a significant change nor were down-regulated in ethanol-fed female mice, suggesting decreased cellular demand for energy in female mice. 3) Female mice fed with alcohol showed up-regulation of cell development process proteins, such as troponin C, troponin I, and myosin light chain. This is consistent with Fogle's report on sex-dependent heart proteomic analysis of alcoholic cardiomyopathy in a rat model.[51] 4) More oxidative stress-related proteins were found to be altered in female mice fed with alcohol. Several studies have reported oxidative stress to be one reason for higher liver injury in female alcoholics.[20], [52] In our study, various oxidative stress-related proteins, such as GSTP1, P4HB, ITIH4 and SOD2, were found to be up-regulated, but others such as MDH1, IDH1 and PRDX6 were found to be down-regulated in females after alcohol feeding as compared to the males. Increased oxidative stress would lead to an increase in the expression of antioxidant enzymes, such as GSTP1 and SOD2.[31], [53] Some antioxidant enzymes, such as PRDX6, were reported to be decreased during oxidative stress, and thus, their reduced expression in alcohol-fed female mice could suggest a state of enhanced oxidative stress.[54] These changes in protein expression are consistent with an increased state of oxidative stress in female mice than that in males after ethanol exposure (Fig. 4A). Other cellular function and hepatotoxicity differences in response to alcohol as indicated by IPA analysis, and other significantly changed proteins between male and female mice are shown in Fig. 4. Together, the data presented herein suggested relative lower metabolic reactions, higher oxidative stress processes and higher cell development processes in the liver of female mice compared to male mice in response to chronic alcohol feeding and these differences may help to clarify the basis of why the female gender is more susceptible to alcohol.

### CYP2E1 knockout has greater effects on liver proteome than that of ethanol and gender

**Figure 5:**
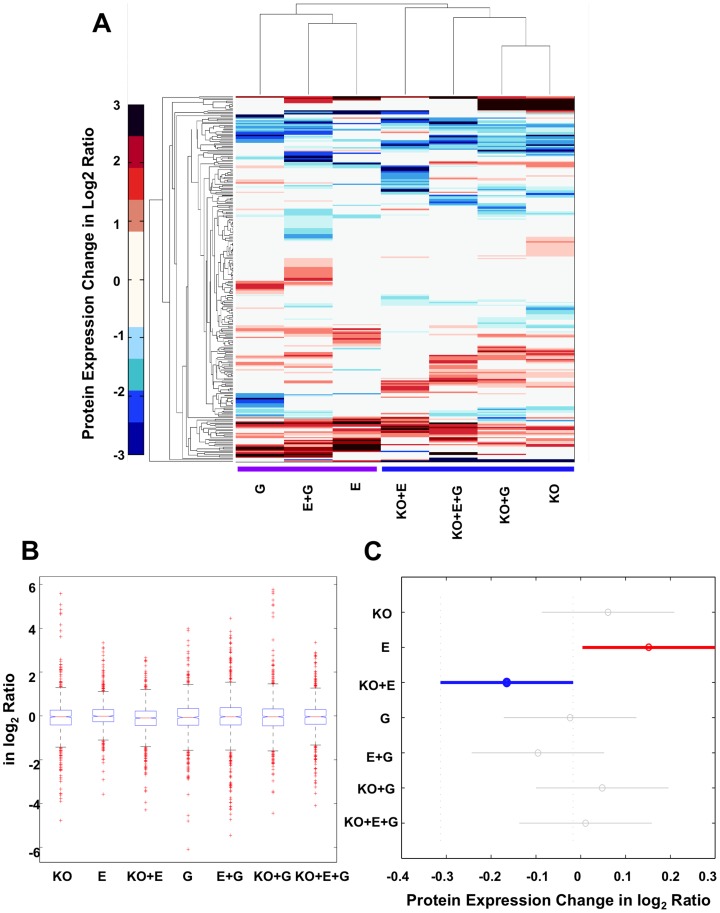
Hierarchical clustering and ANOVA clustering analysis of protein expression changes in the seven observations. (A) Hierarchical clustering analysis. The color bar denotes protein expression change in log2 ratio. Two major clusters were obtained with or without the knockout factor. ANOVA clustering analysis: (B) Boxplot of the protein expression changes in seven observations. The central mark is the median protein expression change in each observation, the edges of the box are the 25th and 75th percentiles, and the whiskers extend to the most extreme data points not considered outliers. The cross mark plotted outlier proteins. (C) Multiple comparisons of the mean protein expression changes in seven observations. This snapshot from the interactive output in Matlab represents the only significantly different observation pair, E and KO+E. KO, CYP2E1 knockout; E, ethanol; G, gender.

To compare the influence of ethanol, CYP2E1 knockout and gender on the mouse liver proteome, we conducted multivariate statistical analysis to the dataset. At first, hierarchical clustering analysis was performed for the seven selected observations on the 270 proteins with p-value less than 0.05 at least in one condition. Based on the prevalence of each observation's features, the seven observations were clustered into two major groups, wild-type and CYP2E1 knockout (Fig. 5A). Meanwhile, the most similar two groups were lined in between observations of ethanol and ethanol plus gender conditions, and between CYP2E1 knockout and CYP2E1 knockout plus gender conditions. The clustering result infers that among the three factors, ethanol, CYP2E1 knockout and gender, the effects of CYP2E1 knockout on global protein expression is greater than that of ethanol and gender.

To further validate that the differences of protein expression level observed in this study were due to the real biological difference between the tested experimental conditions (observations), CYP2E1 knockout, chronic alcohol feeding, and gender, but not the sample or experimental fluctuations, we conducted analysis of variance (ANOVA) with the null hypothesis that the average protein expression was equal across seven selected observations (Fig. 5B). The F test yields p-value of 0.0023, which allowed us to reject the null hypothesis and accept the alternative hypothesis that the average protein expression level was different in at least one observation than the others. In order to find the significantly different observation(s), we conducted a multiple comparison procedure using Tukey-Kramer correction method in Matlab. The only significantly different average protein expression level occurred in conditions between ethanol administration and CYP2E1 knockout plus ethanol administration (Fig. 5C). Again, it confirms that CYP2E1 is the primary factor affecting the global protein expression pattern in the liver under the chronic alcohol feeding condition tested in this study.

The next question we asked was whether only the CYP2E1 knockout without ethanol administration could cause significant change in the liver proteome. Based on previous studies, in normal conditions, there was no difference in fatty acid metabolism and oxidative stress level between dextrose-fed CYP2E1 knockout and dextrose-fed wild-type mice.[16], [34] However, analyzing the proteomic data of protein expression ratio of CYP2E1 knockout male mice fed with dextrose diet (R_KO_, 118/114), 90 proteins were found significantly changed. Among them, 48 proteins were up-regulated and 42 were down-regulated (Table S3). Protein annotation analysis of these 90 proteins indicate that the molecular and cellular functions of these proteins were mainly related to cellular growth and proliferation (12 proteins), oxidation reduction (24 proteins), alcohol, fatty acid and amino acid metabolism (24 proteins), signaling/transcription (9 proteins), energy production (5 proteins), immune response (3 proteins), DNA binding (4 proteins), transport (3 proteins), chaperone (3 proteins), and hypoxia (1 protein).

For many cellular functions, the effect of the CYP2E1 knockout does not always have a strict up- or down- directionality. For instance, in cell glycolysis, we observed increased ALDOB, PGK1 and ALDH2 and decreased FBP1. However, our data has revealed several commonly changed proteins which resulted from both male and female CYP2E1 knockout mice fed the dextrose diet. In the top 10 up- and down-regulated proteins, 6 out of the 10 up-regulated proteins (CKM, MB, MYLPF, TNNI2, TNNT3, and TPM1) found in CYP2E1 knockout male mice were also in the top 10 most significantly increased proteins found in female CYP2E1 knockout mice. Four of these proteins (CKM, MYLPF, TINNI2, TNNT3) were up-regulated by tumor suppressor SMARCA4, which is a SWI/SNF related, matrix associated, actin dependent regulator of chromatin.[55] In the CYP2E1 knockout mice, protein levels of CA3, GPX1, PRDX5 and PRDX6 were increased significantly. These increases, together with CYP2E1 knockout, could result in decreased production of reactive oxygen species. Interestingly, proteins associated with fatty acid transport were significantly increased as reflected by the increased levels of ALB, FABP1, FABP2, FABP3 and SCP2. On the other hand, all detected acyl-CoA-dehydrogenases were significantly decreased in the CYP2E1 knockout mice, including ACADL, ACADM, and ACADVL, which would suggest decreased oxidation of fatty acids[56]. All of these data indicate that in addition to catalyzing metabolism of alcohol, CYP2E1 is also involved in regulation of fatty acid metabolism, energy homeostasis, intracellular oxygen storage and hepatic fibrosis, effects which need to be further investigated.

### CYP2E1 knockout had reversed effect on the ethanol induced oxidative stress and lipid oxidation

**Figure 6:**
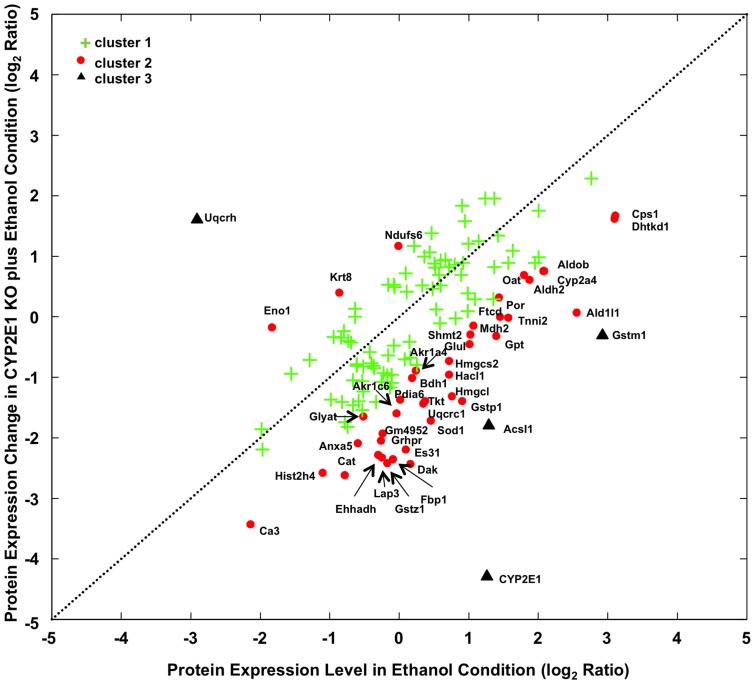
K-mean clustering of proteins with significant expression changes in ethanol and CYP2E1 knockout plus ethanol conditions. The distance for clustering procedure was as described in Methods. Dash line represents where the protein expression change is equal in both conditions. Proteins located in cluster 1 were shown as a green cross, cluster 2 as a red spot and cluster 3 as a black triangle. Proteins located in cluster 2 and 3 were labeled with gene symbols with detailed information in Table 2.

Next we analyzed which proteins significantly altered by CYP2E1 knockout in mice fed with ethanol comparing to wild-type mice fed with ethanol. In another word, what was the CYP2E1 knockout effect on top of ethanol feeding. K-mean clustering is a commonly used method to categorize input data. In K-mean clustering, the algorithm would search for the centric of each cluster and minimize the distance within cluster while maximizing the distance between clusters. The only parameter that needs to be set is the number of clusters one wishes to search. Assuming CYP2E1 had no effect on alcohol fed wild-type mouse, various protein expression levels should not be altered between alcohol-fed wild type and CYP2E1 knockout mice. We defined the distance as the deviation of observed protein expression from equal expression in the two conditions. Therefore the more the protein expression level deviates from equal expression, the more it is affected by CYP2E1 knockout. Proteins with different expression changes (p-value less than 0.05) in at least one condition, either wild-type male mice fed with ethanol (R_E_, 116/114) or CYP2E1 knockout mice fed with ethanol (R_E+KO_, 121/114) were analyzed and found to cluster into three groups (Fig. 6). 82 proteins lied in cluster 1 (green cross) which is close to the expectation line where the protein expression changes in both conditions were not dramatically different. 41 proteins lied in cluster 2 (red spot) and 4 proteins lied in cluster 3 (black triangle) positioned away from the expectation line that indicated the substantial expression change caused by CYP2E1 knockout differ from ethanol feeding alone. As shown in Figure 6, more proteins (three in cluster 3 and 38 in cluster 2) were found in the lower right section which represents the negative effects that CYP2E1 knockout plays on top of ethanol induced protein expression changes. In contrast, only one protein in cluster 3 and three proteins in cluster 2 were localized in the upper left section, where CYP2E1 knockout places positive effects on top of ethanol induced proteins expression changes. Proteins in cluster 2 and 3 are listed in Table 2. GO analysis pointed to oxidative stress and lipid oxidation as the enriched cellular function of these proteins (labeled in Fig. 6). Thus, CYP2E1 knockout had the reversed effect on the ethanol induced oxidative stress and lipid oxidation processes in the liver.

**Table 2:** Proteins with different expression changes in ethanol (116/114) and ethanol plus CYP2E1 knockout (121/114) of cluster 2 and 3 with K-mean analysis.

Accession Number	Protein Name	Gene Symbol	Function	log_2_ (116∶114)	PVal (116∶114)	log_2_ (121∶114)	PVal (121∶114)	Cluster Number
*Proteins analyzed localizing in the lower right section*								
B2KGV2	Carbamoyl-phosphate synthase	Cps1	fatty acid metabolism	3.11	0.008	1.67	0.092	2
**A2ATU0**	**Probable 2-oxoglutarate dehydrogenase E1**	**Dhtkd1**	alcohol metabolism	3.10	0.003	1.62	0.004	2
P10649	Glutathione S-transferase Mu 1	Gstm1	oxidation reduction	2.92	0.000	−0.31	0.967	3
Q8R0Y6	10-formyltetrahydrofolate dehydrogenase	Aldh1l1	oxidation reduction	2.55	0.001	0.07	0.529	2
Q3UER1	Fructose-bisphosphate aldolase	Aldob	alcohol metabolism	2.09	0.000	0.76	0.303	2
Q91XG2	Cytochrome P450, family 2A4	Cyp2a4	oxidation reduction	2.07	0.038	0.76	0.211	2
Q3UKT3	Putative uncharacterized protein	Oat	amino acid metabolism	1.87	0.001	0.61	0.737	2
Q544B1	Aldehyde dehydrogenase 2	Aldh2	alcohol metabolism	1.79	0.000	0.69	0.064	2
**A2A6J8**	**Troponin I, skeletal, fast 2**	**Tnni2**	developmental process	1.57	0.003	−0.01	0.007	2
Q91XD4	Formimidoyltransferase-cyclodeaminase	Ftcd	fatty acid metabolism	1.45	0.005	0.00	0.732	2
P37040	NADPH—cytochrome P450 reductase	Por	oxidation reduction	1.44	0.037	0.32	0.615	2
Q566C3	Alanine aminotransferase 1	Gpt	amino acid metabolism	1.40	0.012	−0.32	0.766	2
**Q6GTG6**	**Long-chain-fatty-acid—CoA ligase 1**	**Acsl1**	fatty acid metabolism	1.29	0.009	−1.79	0.043	3
**Q05421**	**Cytochrome P450 2E1**	**Cyp2e1**	oxidation reduction	1.26	0.001	−4.29	0.034	3
P08249	Malate dehydrogenase	Mdh2	oxidation reduction	1.06	0.024	−0.15	0.571	2
Q9CZN7	Serine hydroxymethyltransferase	Shmt2	fatty acid metabolism	1.02	0.040	−0.29	0.818	2
**P15105**	**Glutamine synthetase**	**Glul**	fatty acid metabolism	1.01	0.001	−0.45	0.045	2
**P19157**	**Glutathione S-transferase P 1**	**Gstp1**	oxidation reduction	0.90	0.011	−1.40	0.000	2
Q8QZS6	3-hydroxy-3-methylglutaryl-Coenzyme A lyase	Hmgcl	fatty acid metabolism	0.76	0.008	−1.32	0.257	2
Q9QXE0	2-hydroxyacyl-CoA lyase 1	Hacl1	fatty acid metabolism	0.72	0.039	−0.96	0.135	2
**P54869**	**Hydroxymethylglutaryl-CoA synthase**	**Hmgcs2**	alcohol metabolism	0.72	0.000	−0.73	0.001	2
P08228	Superoxide dismutase [Cu-Zn]	Sod1	oxidation reduction	0.45	0.938	−1.71	0.001	2
P40142	Transketolase	Tkt	fatty acid metabolism	0.37	0.188	−1.40	0.040	2
Q3TV75	Putative uncharacterized protein	Uqcrc1	oxidation reduction	0.35	0.323	−1.44	0.031	2
Q540D7	Aldehyde reductase	Akr1a4	alcohol metabolism	0.24	0.434	−0.89	0.032	2
Q80XN0	D-beta-hydroxybutyrate dehydrogenase	Bdh1	oxidation reduction	0.19	0.517	−1.01	0.005	2
Q8VC30	FAD-AMP lyase (cyclizing)	Dak	alcohol metabolism	0.16	0.230	−2.43	0.005	2
Q63880	Liver carboxylesterase 31	Es31	alcohol metabolism	0.09	0.959	−2.19	0.001	2
Q3THH1	Putative uncharacterized protein	Pdia6	oxidation reduction	0.01	0.804	−1.37	0.029	2
Q3UEM0	MCG9091, isoform CRA_d	Akr1c6	fatty acid metabolism	−0.04	0.223	−1.59	0.001	2
Q9QXD6	Fructose-1,6-bisphosphatase 1	Fbp1	fatty acid metabolism	−0.09	0.344	−2.35	0.002	2
Q9WVL0	Maleylacetoacetate isomerase	Gstz1	oxidation reduction	−0.17	0.653	−2.42	0.012	2
Q5FW57	Glycine N-acyltransferase-like protein	Gm4952	fatty acid metabolism	−0.24	0.299	−1.93	0.050	2
Q9CPY7	Cytosol aminopeptidase	Lap3	metabolism	−0.25	0.255	−2.33	0.000	2
Q3T9Z2	Glyoxylate reductase	Grhpr	oxidation reduction	−0.27	0.276	−2.05	0.012	2
Q91W49	Enoyl-Coenzyme A, hydratase	Ehhadh	oxidation reduction	−0.31	0.091	−2.29	0.000	2
Q91XE0	Glycine N-acyltransferase	Glyat	fatty acid metabolism	−0.52	0.426	−1.65	0.011	2
P48036	Annexin A5	Anxa5	fatty acid metabolism	−0.60	0.220	−2.09	0.011	2
**Q542K4**	**Catalase**	**Cat**	oxidation reduction	−0.78	0.001	−2.62	0.000	2
B2RTM0	Histone H4	hist2h4	DNA binding	−1.10	0.098	−2.58	0.046	2
**P16015**	**Carbonic anhydrase 3**	**Ca3**	fatty acid metabolism	−2.14	0.000	−3.43	0.000	2
*Proteins analyzed localizing in the upper left section*								
P52503	NADH dehydrogenase iron-sulfur protein 6	Ndufs6	oxidation reduction	−0.01	1.000	1.17	0.048	2
Q58E70	Putative uncharacterized protein	Krt8	developmental process	−0.86	0.046	0.40	0.095	2
Q5FW97	Enolase	Eno1	alcohol metabolism	−1.83	0.000	−0.17	0.056	2
B1ASG5	Ubiquinol-cytochrome c reductase hinge protein	Uqcrh	oxidation reduction	−2.91	0.183	1.61	0.039	3

**Note**: Proteins with significant changes in both conditions are in bold.

### NRF2 and PPARα, upstream key regulators, are involved in ethanol induced oxidative stress and lipid oxidation

**Figure 7:**
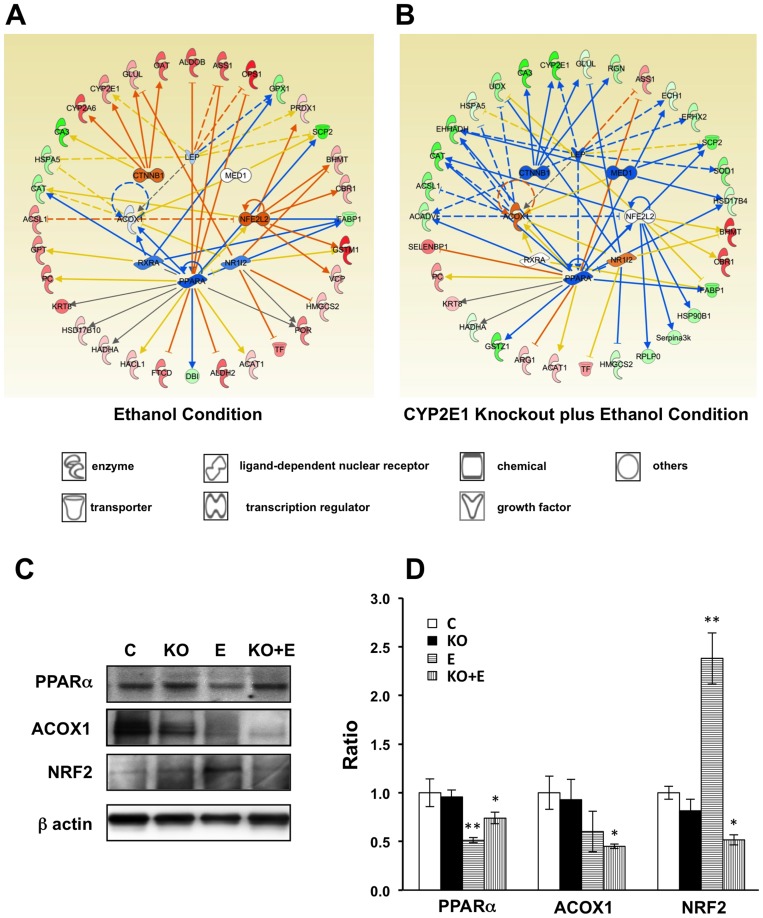
IPA upstream regulator analysis of proteomic data under ethanol and CYP2E1 knockout plus ethanol conditions. Networks and predicted upstream regulators assigned by IPA of differentially expressed proteins in ethanol condition (A) and CYP2E1 knockout plus ethanol condition (B). Symbols of target proteins in red color indicated the increase while in green color indicated the decrease in abundance. Symbol of upstream regulators in orange color indicated the predicted activation while in blue color indicated the predicted inhibition in confidence. The color intensity corresponds to the degree of significance. Proteins in white are those identified through the IPA Knowledge Base. Solid line indicates a direct molecular interaction, and a dashed line indicates an indirect molecular interaction. The orange, blue, yellow and gray lines indicated the predicted relationships as leading to activation, inhibition, finding inconsistent with state of downstream molecule, and effects not predicted, respectively. The symbol shapes denoted the molecular classes of the proteins. Western blot analysis of PPARa, ACOX1 and NRF2 (C, D). All values presented as the mean ±SD of the four mice in each group that have been normalized to β-actin and relative to control mice (Dextrose diet, wild-type, male mice). CON, control; KO, CYP2E1 knockout; E, ethanol. *p<0.05 and **p<0.01.

To further study the “protective” role of CYP2E1 knockout on ethanol induced oxidative stress and lipid oxidation processes in the liver i.e. failure of ethanol to induce oxidative stress and steatosis in the CYP2E1 knockout mice, and to investigate the pathways that play key roles in these processes, IPA was adopted for network and upstream regulator analysis within the two conditions, wild-type mice fed with ethanol (R_E_, 116/114) and CYP2E1 KO mice fed with ethanol (R_E+KO_, 121/114). Proteins in above K-mean clusters 2 and 3 with significant expression changes (p-value less than 0.05) were analyzed against an IPA gene expression database. The predicted upstream regulator activation and inhibition were measured by activation z-scores and the significance indicated by p-value of overlap. 48 upstream regulators were reported, in which eight regulators with activation scores are listed in Table 3 (detailed information in Table S4). The predicted gene expression activations and inhibitions of these eight upstream regulators in wild-type male mice fed with ethanol and CYP2E1 knockout male mice fed with ethanol are illustrated in Figure 7A and 7B, respectively. Notably, the top two proteins NRF2 (also known as NFE2L2 shown in Fig. 7, Table 3 and Table S4) and PPARα were predicted to be activated and inhibited with activation scores of 2.560 (p value 4.76×10^−4^) and −2.309 (p value 6.51×10^−14^) under the chronic ethanol condition based on the observed proteomic data, respectively. Under the chronic ethanol feeding condition (R_E_, 116/114), changes of 7 “target” molecules (VCP, PRDX1, GSTP1, GSTM1, FABP1, CBR1 and BHMT) were consistent with the activation of NRF2 (Fig. 7A and Table 3). NRF2, as a critical transcription factor, plays a central role in regulating both constitutive and inducible expression of a wide variety of antioxidant and anti-inflammatory genes in mammalian tissue and cells.[57]–[59] Lamle *et al* reported that ethanol induced liver injury was more severe in NRF2 knockout mice compared to WT mice, suggesting that NRF2 protects against alcohol liver disease.[60] As shown in Fig. 7A, VCP, PRDX1, GSTM1, CBR1 and BHMT, proteins whose genes are known to be up-regulated by NRF2 are all increased, and FABP1, known to be down-regulated by NRF2, is decreased in the dataset of 116/114. Studies have demonstrated that polymorphisms of GST, especially GSTM1, were associated with an increased risk of developing alcohol liver disease.[61], [62] These findings are consistent with the notion that GST enzymes play an important role in the detoxification of reactive aldehydes, including those that participate in alcohol induced liver injury. Therefore, the upstream transcriptional regulator NRF2 was predicted to be activated under chronic ethanol feeding. The induction of antioxidant and anti-inflammatory enzymes via the NRF2 signaling pathway might act as an early compensatory or adaptive mechanism to suppress ethanol induced oxidative injury.

**Table 3:** IPA upstream regulator analysis of proteins in ethanol (116/114) and ethanol plus CYP2E1 knockout (121/114) conditions.

Upstream Regulator	Molecular Type	Predicted Activation State		Activation z-Score		p-Value of Overlap		Target Molecules in Dataset	
		116/114	121/114	116/114	121/114	116/114	121/114	116/114	121/114
NRF2	transcription regulator	activated	−	2.56	−0.03	4.76E-04	5.46E-04	**Bhmt**, **Cbr1**, *Fabp1*, **Gstm1**, **Gstp1**, **Prdx1**, **Vcp**	**Bhmt**, **Cbr1**, *Fabp1*, *Gstp1*, *Hsp90b1*, *Rplp0, Serpina3k, Uox*
PPARa	ligand-dependent nuclear receptor	inhibited	inhibited	−2.31	−2.29	6.41E-14	1.45E-10	**Acat1**, *Acox1*, **Aldh2**, **Aldob**, **Ass1**, *Cat*, **Cps1**, *Dbi, Fabp1*, **Ftcd**, **Gstp1**, **Hacl1**, **Hadha**, **Hsd17b10**, **Krt8**, **Pc**, **Por**, *Scp2*	*Acadvl*, **Acat1**, *Acox1*, **Arg1**, **Ass1**, *Cat, Ech1, Ehhadh, Fabp1, Gstp1*, *Gstz1, Hadha, Hsd17b4*, **Krt8, Pc**, *Scp2*, **Selenbp1**
CTNNB1	transcription regulator	−	−	1.50	−1.96	3.52E-06	2.75E-04	*Ca3*, **Cyp2a6**, **Cyp2e1**, **Glul**, **Oat**	*Ca3*, *Cyp2e1*, *Glul*, *Rgn*
ACOX1	enzyme	−	−	−	1.70	3.73E-05	4.14E-09	*Acox1*, **Acsl1**, *Cat*, *Hspa5*	*Acadvl*, *Acox1*, *Acsl1*, *Cat*, *Ehhadh*, *Hspa5, Uox*
RXRA	ligand-dependent nuclear receptor	−	−	−1.13	−	7.84E-05	3.84E-02	*Acox1*, *Fabp1*, **Gpt**, *Gpx1*	*Acox1*, *Fabp1*
NR1I2	ligand-dependent nuclear receptor	−	−	−1.03	1.20	1.04E-06	8.37E-05	*Acox1*, **Bhmt**, **Glul**, **Gstm1**, **Gstp1**, **Hmgcs2**, **Po**r, **Tf**	*Acox1*, **Bhmt**, *Glul*, *Gstp1*, *Hmgcs2*, **Tf**, *Uox*
MED1	transcription regulator	−	inhibited	−	−2.21	5.41E-04	1.90E-06	*Acox1*, **Gstp1**, *Scp2*	*Acox1*, *Ehhadh*, *Gstp1*, *Hsd17b4*, *Scp2*
LEP	growth factor	−	inhibited	−0.38	−2.12	8.94E-07	7.89E-07	*Acox1*, **Aldob**, **Ass1**, **Cps1**, **Cyp2e1**, *Gpx1*, **Prdx1**, *Scp2*	*Acadvl*, *Acox1*, **Ass1**, *Cyp2e1*, *Ech1*, *Ehhadh, Ephx2, Scp2, Sod1*

**Note**: Proteins with up-regulated changes are displayed in bold, and with down-regulated changes are displayed in italic.

Meanwhile, 18 proteins were identified as “target” molecules of PPARα (Table 3) and the changes of 11 of these proteins (SCP2, GSTP1, FTCD, FABP1, DBI, CPS1, CAT, ASS1, ALDOB, ALDH2 and ACOX1) were consistent with the inhibition of PPARα (Fig. 7A). The inhibition of PPARα by ethanol, a master regulator of lipolysis, with a subsequent decrease in lipolytic enzymes such as acyl-CoA oxidase 1 (ACOX1) and increase in lipogenesis proteins such as Acyl-CoA synthetase long-chain family member 1 (ACSL1) would result in a fat-storing metabolic remodeling of the liver and thereby could play a key role in the overall mechanism of ethanol induced fatty liver.[63] Furthermore, the decreases of two major enzymatic antioxidants, catalase (CAT) and glutathione peroxidase 1 (GPX1), would be the result of PPARα pathway inhibition after ethanol feeding through the intermediate regulators ACOX1 and retinoid X receptor, alpha (RXRA), respectively.

Cadherin-associated protein beta1 (CTNNB1), another upstream “master” regulator, was predicted to be activated with an activation score of 1.498 (p value 3.52×10^−6^). It was indicated as the “master” regulator of CYP2E1 (shown in Fig. 7A) in the network analysis. This regulation was reported by previous studies, as the loss of CYP2E1 makes CTNNB1 knockout mice resistant to acetaminophen-induced hepatotoxicity.[64], [65] Meanwhile, CTNNB1 is also required for CYP1A2 and CYP2E1 expression.[64]

In comparison, with the “target” molecules expression changes in CYP2E1 KO mice fed with ethanol (R_KO+E_, 121/114, shown in Fig. 7B), the predicted activation scores of NRF2 and CTNNB1 became negative, which indicates inhibition of these two genes as well as for MED1. The predicted activation scores of ACOX1 and NR1I2 became positive, which indicates activation (Table 3). The predicted activation score of RXRA changed from a negative value to almost neutral. All of these above facts indicate that CYP2E1 KO is overwriting some of the effects of ethanol on the upstream regulators or that the effects of ethanol on the upstream regulators require CYP2E1. But the predicted activation score of PPARα remained almost the same under both conditions (Table 3). This suggests that PPARα regulation was not affected by CYP2E1. The upstream regulator analysis also illustrated in addition to the 8 upstream regulators, there might be others involved in some of the downstream targets, such as BHMT, CBR1, and TF. These three proteins (increased in proteomic data set) were linked to NRF2 under chronic ethanol conditions but were decoupled under CYP2E1 KO with chronic ethanol conditions. The upstream regulator analysis could also indicate the primary upstream regulators when a downstream “target” protein was regulated by more than two “master” regulators. For example, the liver form of fatty acid-binding protein (FABP1) was regulated by PPARα, NRF2 and RXRA. Under chronic ethanol conditions, decrease of FABP1 is linked to activation of NRF2 and inhibitions of PPARα and RXRA. However, under CYP2E1 KO with chronic ethanol condition, the proteomic data only matched to the inhibition of PPARα and decoupled with NRF2 and RXRA. This analysis predicted that the FABP1 was mainly regulated by PPARα. FABP1 and sterol carrier protein-2 (SCP2) have been reported as being regulated by PPARα and reduced in alcohol induced liver injury.[66]

The IPA analysis results were further confirmed with Western blot analysis (Fig. 7C). The level of PPARα was not affected by the CYP2E1 KO. It was significantly decreased by chronic ethanol feeding in wild-type and CYP2E1 KO mice (Fig. 7D). Similarly, the level of ACOX1 was also decreased by chronic ethanol feeding but was only significant in CYP2E1 KO mice (Fig. 7D), which is consistent with the proteomic data (Table S1). However, the Western blot and proteomic data were opposite with the predicted results of IPA upstream regulator analysis. This discrepancy might indicate that in the IPA analysis we need to be more cautious for the indirect relationships (shown as dotted lines in Fig. 7A and B), because there might be missing regulators. We should also consider that the IPA upstream regulator analysis was based on RNA expression data. The difference might reflect the differences between transcriptional regulation and protein regulation. Lastly, examining those direct interactions, the level of NRF2 was augmented after chronic ethanol feeding in wild-type mice but decreased in CYP2E1 knockout mice fed with ethanol (Fig. 7D), which is in agreement with the predictions of IPA upstream regulator analysis.

### Conclusion

An integrated quantitative proteomics platform was developed and applied to understand ethanol, CYP2E1 and gender effects on the liver proteome and on alcohol induced liver injuries. Through this platform, we obtained a multifactorial comparative data set of mouse liver protein expressions. Subsequent systematic multivariant data analyses and network analysis allowed us to discover novel relationships among these multiple conditions and therefore identify important proteomic changes with meaningful information, at the protein level. These key pieces of information helped in understanding chronic ethanol feeding induced liver damage associated with different responses of gender and CYP2E1's effects.

## Supporting Information

Table S1 **Protein report of 878 identified proteins generated from ProteinPilot with detailed identification and quantification information using 114 and 113 as denominators.**
(XLSX)

Table S2 **Proteins significantly changed after ethanol administration in male and female mice.**
(XLSX)

Table S3 **Proteins significantly changed in CYP2E1 knockout dextrose-fed male mice.**
(XLSX)

Table S4 **IPA upstream regulator analysis of proteins in clusters 2 and 3 resulted from K-mean analysis of log2 R_E_, 116/114 and log2 R_KO+E_, 121/114 data sets.**
(XLSX)
